# Quality-by-Design Optimization of Mucoadhesive Trimethyl Chitosan-Coated Alginate/Dextran Sulfate Nanoparticles for Oral Insulin Delivery

**DOI:** 10.3390/md24060196

**Published:** 2026-06-01

**Authors:** Bruno Pessoa, Daniel Vanzan, Lucio Cabral, Antonio J. Ribeiro

**Affiliations:** 1 Faculty of Pharmacy, University of Coimbra, 3000-548 Coimbra, Portugal; 2 Faculty of Pharmacy, Federal University of Rio de Janeiro, Rio de Janeiro 21941-902, Brazil; 3 Genetics of Cognitive Disfunction, i3S, Instituto de Biologia Molecular e Celular (IBMC), Rua Alfredo Allen, 4169-007 Porto, Portugal

**Keywords:** alginate, dextran sulfate, trimethyl chitosan, oral insulin delivery, mucoadhesion, quality by design, nanoparticles

## Abstract

Trimethyl chitosan (TMC)-coated alginate/dextran sulfate (ADS) nanoparticles were developed as mucoadhesive nanocarriers for oral insulin delivery using a Quality-by-Design strategy. In a first screening step, a two-level factorial design was applied to evaluate the influence of ADS concentration, TMC concentration, insulin concentration, and poloxamer^®^ concentration on particle size and encapsulation efficiency. The screening design identified the ADS-TMC pair as the main formulation parameter for particle size, while TMC and poloxamer^®^ were the most influential factors for encapsulation efficiency. In a second step, formulation optimization was performed using a three-factor, three-level Box–Behnken design in which ADS concentration, TMC concentration, and the degree of quaternization (DQ) of TMC were investigated as critical material attributes. Particle size, zeta potential, and in vitro mucoadhesion were selected as critical quality attributes. Across the Box–Behnken design, the experimental formulations showed particle sizes ranging from 316 to 1340 nm, zeta potentials between +17 and +39 mV, and mucin-binding values from 7 to 87%. Numerical optimization by Design-Expert^®^ desirability analysis identified an optimal formulation composed of 0.096% (*w*/*v*) ADS and 0.700% (*w*/*v*) TMC with 60% DQ. The model predicted a particle size of 316.24 nm, a zeta potential of +38.43 mV, and an in vitro mucoadhesion of 87.14%. Experimental confirmation yielded values of 330.79 nm, +37.09 mV, and 84.61%, respectively, with prediction errors below 5% for all responses. In simulated gastric medium, partial insulin leakage was observed during the first 120 min, whereas cumulative insulin release reached 54% after 5 h in simulated intestinal medium. These results demonstrate the usefulness of a QbD framework combined with desirability-based optimization for defining robust formulation conditions for mucoadhesive TMC-coated ADS nanoparticles intended for oral insulin delivery.

## 1. Introduction

Oral delivery of insulin remains difficult because the peptide must remain stable during processing, survive the acidic and enzymatic environment of the gastrointestinal (GI) tract, and subsequently interact with the intestinal mucosa in a form suitable for absorption [1]. Nevertheless, oral insulin remains attractive because it is non-invasive, may improve patient acceptance and adherence, and could partially reproduce the physiological portal route of endogenous insulin delivery before systemic distribution. Biopolymer-based nanoparticles are attractive for this purpose because their composition can be adjusted to combine peptide protection, controlled release, and mucosal interaction within the same carrier. However, these functions depend strongly on the architecture of the formulation, particularly particle size, surface charge, colloidal stability, and the balance between drug retention and release [2,3,4].

Polysaccharide-based systems are especially useful because oppositely charged polymers can form mild polyelectrolyte complexes without exposing insulin to harsh organic solvents or aggressive processing conditions. In ADS-based nanoparticles, alginate and dextran sulfate provide a negatively charged matrix capable of interacting with insulin and cationic coating polymers [5,6].

Alginate is an anionic copolymer of mannuronic and guluronic acid residues that forms ionically crosslinked hydrogels with divalent cations such as Ca^2+^ and Zn^2+^ [7]. Dextran sulfate is a highly sulfated branched polysaccharide with a high density of negative charges, which interacts strongly with polycations and contributes to the formation of dense polyelectrolyte networks [8]. Chitosan, derived from the deacetylation of chitin, is a linear chain composed of glucosamine and N-acetylglucosamine monomer units, and besides its biodegradability and biocompatibility, it becomes positively charged under acidic to mildly acidic conditions [9,10]. Taken together, alginate and marine-sourced chitosan can be viewed as complementary marine biomaterials, since they combine renewable marine origin with physicochemical properties that favor hydrogel formation, polyelectrolyte complexation, and nanostructure assembly for drug-delivery applications.

In ADS systems, chitosan stabilizes the hydrogel core during nanoparticle formation, strengthens the polyanionic network, and contributes to insulin retention via electrostatic interactions [7,9]. Moreover, chitosan is a well-characterized mucoadhesive and absorption-enhancing polymer, as its protonated amino groups can interact with oppositely charged mucin and transiently open tight junctions, thereby promoting paracellular transport of large-molecular-weight molecules across the intestinal epithelium [11].

In such nanoparticles, the structure and composition of the outer anionic/polysaccharide shell are critical for achieving the desired balance between insulin protection, release, and interaction with the intestinal mucus layer. Previous studies have shown that, among the various components, the amount and characteristics of chitosan exert a pronounced effect on the main nanoparticle’s physicochemical properties, including insulin release [6,12,13]. However, chitosan as a biopolymer is one of the most difficult polymers to standardize, as its molecular weight and degree of acetylation can vary between batches, influencing solution conformation, complexation ability, and mucoadhesive behavior [11,14]. Together with the increasing demand for comprehensive nanoparticle characterization [15,16], disclosure of chitosan attributes is critical for a better understanding of the contribution of the marine biopolymer to nanoparticle properties.

Another limitation of chitosan is its pH-dependent solubility and ionization. As the pH approaches neutrality, chitosan becomes less protonated and less soluble, which may reduce its mucoadhesive and permeation-enhancing effects in the small intestine, where insulin absorption is expected to occur [9]. Quaternized derivatives such as N-trimethyl chitosan (TMC) have been developed to overcome these limitations. TMC remains soluble and positively charged over a broader pH range and has been reported to increase both mucoadhesion and paracellular transport relative to native chitosan [9,17]. Substituting chitosan with TMC in multilayer ADS nanoparticles is therefore expected to alter nanoparticle properties, including their surface charge, colloidal stability, and interaction with mucus behavior, with potential benefits for oral insulin delivery. However, the effect of this substitution on the mucoadhesive properties of such nanoparticles has not yet been systematically investigated.

Although TMC-containing insulin nanocarriers have shown promising preclinical behavior, most recent studies have focused on more complex multifunctional systems, hybrid coatings, or alternative oral-delivery technologies rather than on the systematic study of the simpler ADS-TMC interface [18]. Therefore, the novelty of the present work does not lie in the general use of TMC- or alginate-based insulin carriers, which have been previously reported, but in the Quality-by-Design-guided optimization of ADS/TMC nanoparticles, with explicit consideration of TMC degree of quaternization and mucoadhesion as formulation-driving quality attributes. This approach allows a more rational understanding of how TMC chemistry and polymer balance control nanoparticle performance.

**Figure 1:**
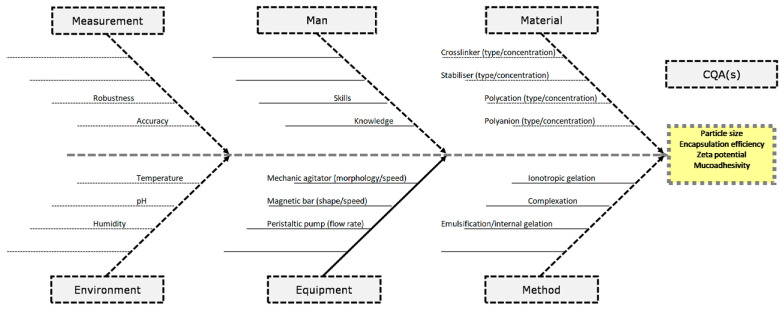
Ishikawa diagram summarizing the main formulation, process, environmental, and analytical variables that may influence the critical quality attributes of TMC-coated ADS nanoparticles intended for oral insulin delivery.

Nanoparticle formulations composed of biopolymers such as alginate, dextran sulfate, and TMC involve several formulation and process variables that may interact with each other (Figure 1). In the Ishikawa diagram, the Material branch includes the chemical attributes of the polymers, drug, crosslinker, and stabilizer, which directly affect electrostatic complexation, particle formation, and insulin retention. The Method branch reflects critical preparation steps such as ionotropic gelation and polyelectrolyte complexation, which govern core formation and coating efficiency. The Equipment branch includes stirring and pumping conditions that may influence mixing and particle formation, whereas the Environment branch includes pH and temperature, which can alter polymer ionization and colloidal stability. Finally, the Measurement and Operator branches reflect analytical robustness and handling variability, both of which may affect the reproducibility of the final CQAs. Among these variables, the polymer, drug, and surfactant concentrations were selected as critical factors for the present study based on literature data [19,20] and preliminary process knowledge.

Multiparametric experimental design is a powerful tool to evaluate the impact of these variables on CQAs and to identify optimal operating conditions [21]. Design of experiments (DoE) approaches have been successfully applied to nanoparticle systems to reduce the number of experimental runs, build predictive models, and reveal possible factor interactions [22,23]. In an optimization design, the response is typically described by a second-order polynomial, allowing assessment of linear, interaction, and quadratic effects [23].

Within this context, this study builds on the established ADS insulin nanoparticle platform and focuses on the role of the cationic coating polymer. The main objective is to evaluate the effect of TMC coating of ADS cores on the physicochemical characteristics and mucoadhesive behavior of insulin-loaded nanoparticles. To evaluate the influence of ADS concentration, TMC concentration, and TMC degree of quaternization on particle size, zeta potential, and quantitative indices of mucoadhesion, a three-factor, three-level Box–Behnken design and response surface methodology were used. The novelty of this work lies in the multiparametric optimization of TMC-based coatings for nanocarriers formed from an ADS polyanionic blend intended for oral insulin delivery, with a specific emphasis on enhancing and better controlling mucoadhesive interactions at the intestinal mucosa.

## 2. Results

Pregel cores made of alginate, dextran sulfate, poloxamer^®^ 188, and insulin were coated with TMC and PEG to deliver insulin by the oral route. TMCs with different degrees of quaternization were successfully synthesized and characterized before the preparation of nanoparticles. The resulting nanoparticles were first submitted to a physicochemical characterization, then to an evaluation of insulin in vitro release testing from nanoparticles, and an assessment of an in vitro bioactivity evaluation of the peptide drug. Finally, the nanoparticle formulation was optimized using a QbD approach.

### 2.1. Characterization of TMC

**Figure 2:**
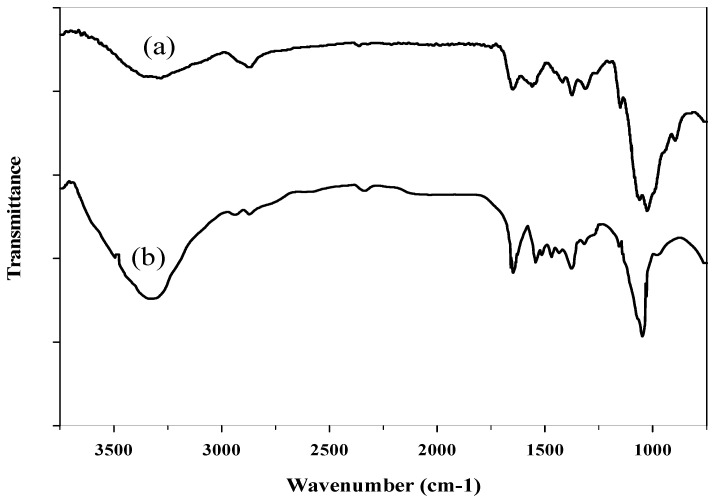
FTIR spectra of CHI (**a**) and TMC (**b**).

The successful methylation of chitosan was confirmed by FTIR and ^1^H NMR. The FTIR spectra of chitosan and the synthesized TMC are presented in Figure 2. The band observed at around 1650 cm^−1^ corresponds to the amide I vibration of the N-acetylated units in chitosan [24]. The signal near 1555 cm^−1^ is associated with N–H bending of mono- and disubstituted amino groups in both chitosan and TMC. In addition, a broad absorption band centered at approximately 3436 cm^−1^ appears in both spectra and is attributed to the overlapping stretching vibrations of –OH and –NH_2_ groups [25]. A new band emerging at about 1478 cm^−1^ is assigned to the asymmetric C–H stretching of methyl groups, which is characteristic of quaternized, methylated chitosan salts [24,26]. This feature confirms the presence of partially methylated chitosan species (for example, dimethylated chitosan) in the TMC sample.

**Figure 3:**
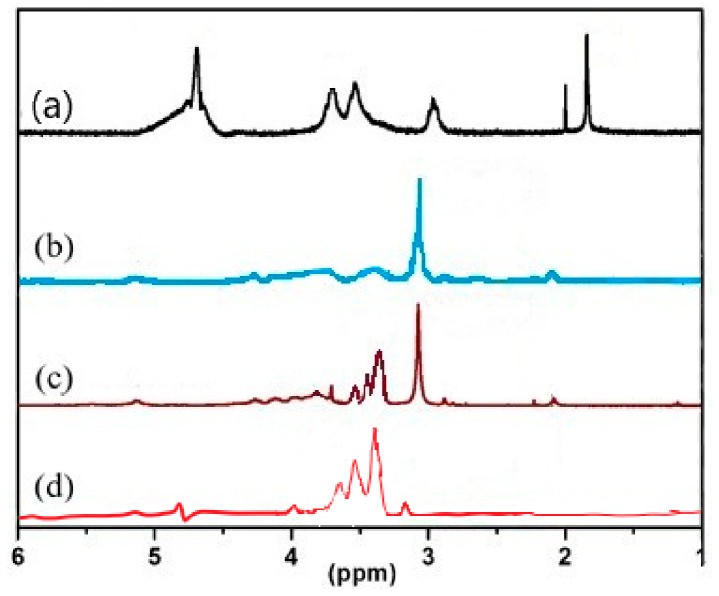
^1^H NMR spectra of CHI (**a**), TMC20 (**b**), TMC40 (**c**), and TMC60 (**d**).

The ^1^H NMR results further confirmed that chitosan was successfully methylated. In the ^1^H NMR spectrum (Figure 3), the signals between approximately 3.4 and 4.7 ppm are attributed to the saccharidic protons of the chitosan backbone (H_5_, H_6_, H_3_, H_4_ and H_1_), while the resonances appearing at around 3.2 ppm and 2.6 ppm correspond to the methyl protons of the quaternary –N^+^(CH_3_)_3_ and the tertiary –N(CH_3_)_2_ groups, respectively, confirming quaternization of the amino groups [27]. The ratio of the integrated areas of these methyl signals was used to calculate the average DQ of TMC20, TMC40, and TMC60 samples, which were found to be 21.4%, 38.9%, and 58.8%, respectively. Although higher levels of methylation can be obtained by extending the reaction, the harsh alkaline and high temperature conditions also promote degradation of the chitosan backbone [28], which reduces molecular weight and may adversely affect the material’s functional properties [29]. In the present work, a chitosan derivative with improved solubility (TMC) was obtained using a relatively short reaction time, allowing the molecular weight of the polymer to be largely preserved.

**Figure A1:**
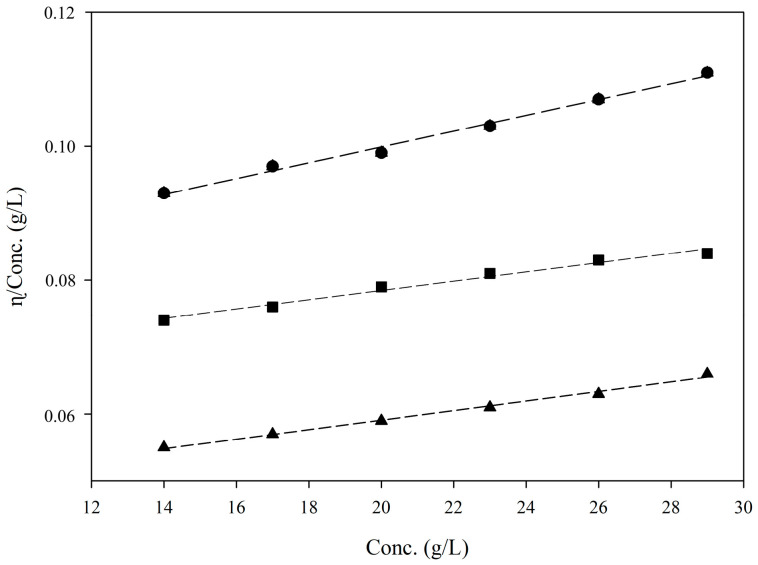
Intrinsic viscosity of TMC20 (●), TMC40 (■), and TMC60 (▲).

An inverse relationship between intrinsic viscosity and the DQ of the chitosan derivatives was observed (Figure A1 in Appendix A). Such behavior has been described for TMC systems, in which increasing DQ is frequently associated with a decrease in molecular weight. While methylation increases the mass of individual repeating units, the basic and elevated-temperature conditions typically used for reductive methylation can induce partial cleavage of the polymer backbone, so that the net result is a lower average molecular weight and reduced intrinsic viscosity. In agreement with the present data, a stepwise decrease in both molecular weight and intrinsic viscosity from chitosan to trimethylated derivatives has been reported [30,31], and recent reviews on quaternized chitosan summarize the same tendency for highly quaternized TMC derivatives [32].

### 2.2. Physicochemical Characterization of TMC Nanoparticles

**Figure 4:**
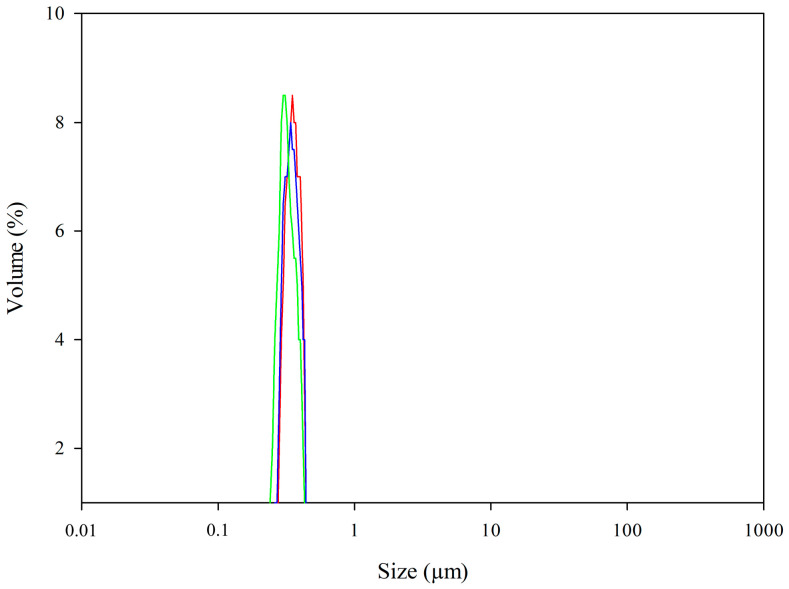
Granulometric profile of ADS nanoparticles coated with TMC obtained by laser diffraction. The formulation contained ADS 0.05% (*w*/*v*), TMC 0.3% (*w*/*v*), insulin 0.008% (*w*/*v*), and poloxamer^®^ 0.03% (*w*/*v*). Measurements were performed on three independently prepared batches. No micrometric population was detected within the measurement range of this analysis.

Nanoparticle size was assessed by dynamic light scattering, whereas laser diffraction was used as a complementary method to screen for the possible presence of larger particle populations. The mean particle size of the TMC-coated nanoparticles was 387 ± 31 nm, calculated from measurements obtained for three independently prepared batches, with a PDI of 0.40 ± 0.04. Although these values are somewhat higher than those reported for some trimethyl-coated alginate nanoparticles [33], differences in chitosan properties limit direct comparison among studies. As can be seen in Figure 4, laser diffraction analysis did not reveal a detectable micrometric particle population within the measurement range of the method.

**Figure 5:**
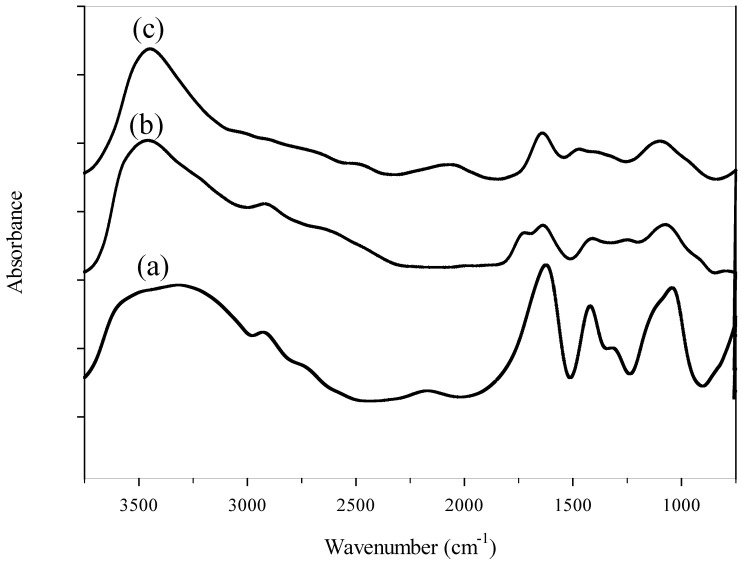
FTIR spectra of ADS (**a**), TMC (**b**), and TMC-coated ADS nanoparticles (**c**).

FTIR spectra of the formulations are shown in Figure 5. All spectra were plotted over the same experimentally accessible range of 750–3750 cm^−1^. The broad band observed in all FTIR spectra between 2360 and 3720 cm^−1^ was assigned to the characteristic C-H, N-H, and O-H stretches. The band at 1482 cm^−1^ (spectrum c) was attributed to the expected angular deformation of C-H bonds of the N-methyl group-exhibiting TMC structure [34], whereas the band at 1642 cm^−1^ observed for TMC-coated nanoparticles (spectrum b) was attributed to C=O bonds of secondary amide groups, arising from acetylated residues that remain in the TMC structure and from axial asymmetric deformation of carboxylates. The band observed at approximately 1420 cm^−1^ is attributed to the symmetric stretching of carboxylate groups [35]. In the nanoparticle spectrum, this band appears with lower intensity than in the ADS spectrum (spectrum a), which can be explained by partial protonation of the alginate carboxylate groups and by their participation, together with dextran sulfate groups, in electrostatic complexation with the positively charged sites of TMC. Although the full 400–750 cm^−1^ fingerprint region was not recorded, the lower-wavenumber portion that was experimentally accessible was retained for all samples and discussed only in relation to the most informative polysaccharide bands.

### 2.3. In Vitro Release and Bioactivity of Insulin

**Figure 6:**
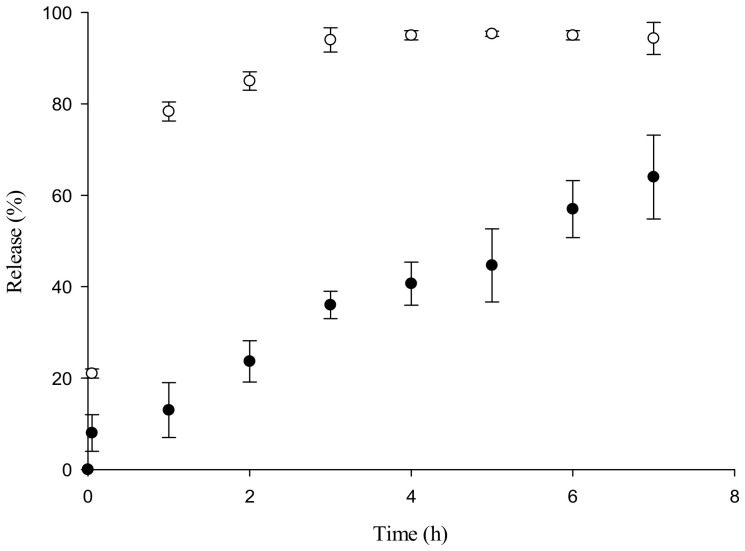
Insulin releasing profile from nanoparticle formulation coated with TMC at 0.3% (*w*/*v*) (•). Free insulin (ο) was used as a control, and all results presented are the mean of three replicates.

Although insulin release was not included as a CQA in the optimization workflow, it was evaluated as a proof-of-concept functional test using a representative TMC-coated formulation selected from the initial screening stage. This formulation was chosen because it displayed submicron size and no detectable micrometric population by laser diffraction, making it suitable for an initial assessment of pH-dependent insulin release. As seen in Figure 6, in simulated gastric medium, partial insulin leakage was observed during the first 120 min, indicating that the polymeric network did not completely prevent peptide diffusion under acidic conditions. This behavior may result from competition between the positively charged TMC coating and insulin for interaction with the negatively charged ADS matrix, which can reduce the strength of insulin retention within the core [33]. After transfer to simulated intestinal medium, cumulative release reached 54% after 5 h. This pH-triggered release profile suggests that the formulation can retain a relevant fraction of insulin during gastric exposure while allowing peptide availability under intestinal conditions, close to the expected absorption site [1].

**Figure 7:**
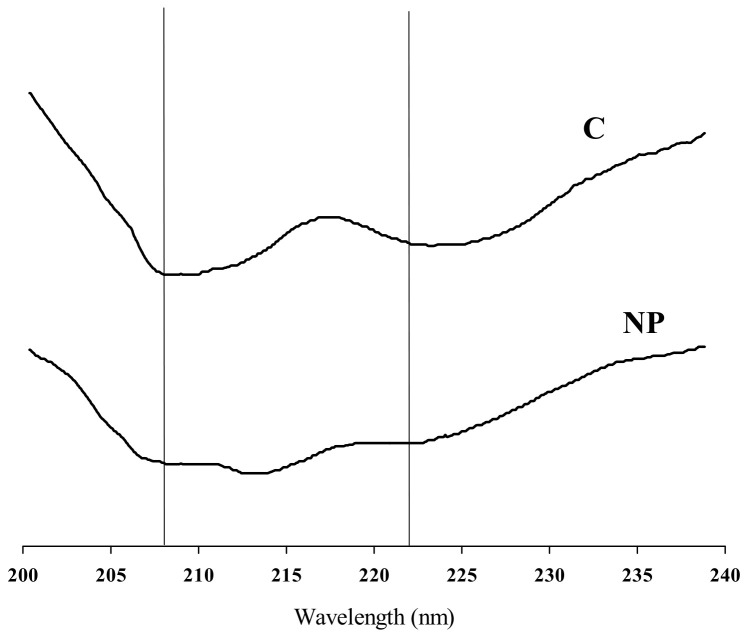
Circular dichroism (CD) spectra of insulin in solution (10 µM) in phosphate-buffered saline (PBS) at pH 7.4 and 25 °C: (C) control (unprocessed insulin), (NP) insulin released from TMC nanoparticles.

Circular dichroism, used here to monitor the conformational integrity of insulin (Figure 7), showed that the spectrum of unprocessed insulin in PBS at pH 7.4 (control, C) exhibits two negative bands at approximately 209 and 224 nm, which is characteristic of a predominantly α-helical structure with some β-sheet contribution. The spectrum of insulin recovered from the nanoparticles (NP) displayed a similar overall shape, although the intensity of these minima was slightly reduced compared with the standard solution. This suggests that the secondary structure of insulin undergoes only minor perturbations upon encapsulation and release. A plausible explanation is that insulin interacts with the surrounding biopolymers, leading to subtle changes in its environment and conformation, but without causing denaturation or loss of bioactivity, so that the essential secondary structure is preserved after exposure to the release medium [36].

### 2.4. Experimental Design: Exploratory Experiments

A wide range of variables can influence the critical quality attributes of nanoparticles. As a first step, exploratory experiments were carried out to identify the process parameters that most strongly affected the particle properties of interest [37,38], including the size of ADS nuclei, stirring during coating, and polycation addition flow rate. Coating ADS gel cores with TMC at 0.03% (*w/v*) at a stirring rate lower than 800 rpm, nanoparticles with a size of more than 1000 nm were obtained, and increasing the speed up to 1300 rpm produced smaller-diameter nanoparticles. Beyond 1300 rpm, stable and reproducible stirring under experimental conditions was no longer possible. Regarding the effect of the polycation addition rate, an addition rate of 1.6 mL/min could promote more uniform mixing before extensive complex growth, often yielding smaller, less aggregated microgel complexes. Interaction with mixing intensity in oppositely charged polyelectrolytes is critical: stirring and polycation addition rate are not independent in practice. They further interpret shrinkage as plausibly driven by chitosan diffusion into the alginate matrix and partial network collapse due to electrostatic neutralization, noting diffusion is enhanced at higher mixing rates. Consequently, stirring at 1300 rpm and a polycation addition rate of 1.6 mL/min were used in subsequent experiments.

### 2.5. Criticality Assessment of Material Input Factors

Insulin-loaded ADS cores were successfully coated with TMC in the presence of poloxamer^®^, producing nanoparticles with mean sizes between 218 and 473 nm and encapsulation efficiencies between 44 and 84% (Table 1). The two-level, four-factor screening design allowed the preliminary identification of the formulation variables with the greatest impact on the selected CQAs, namely particle size and encapsulation efficiency. The model indicated that particle size was mainly governed by the combined effect of ADS and TMC, rather than by either factor alone. This finding supports the central role of the ADS-TMC polyelectrolyte balance during the coating step. As ADS provides the anionic core and TMC supplies the cationic coating, their relative proportion determines the extent of charge compensation, shell compaction, and possible particle aggregation. Within the experimental domain studied, increasing TMC concentration contributed to particle size reduction, suggesting that TMC promoted condensation of the ADS cores when present in a suitable ratio. This behavior is consistent with reports on alginate/chitosan and alginate/TMC systems, where nanoparticle size is strongly dependent on the anionic/cationic polymer ratio, and smallest particles are generally obtained within an optimal complexation window [7,33,39]. For EE, the most relevant factors were TMC concentration and poloxamer^®^ concentration. TMC showed a positive effect on EE, indicating that increasing the amount of cationic polymer favored insulin association with the nanoparticle matrix. This can be attributed to stronger electrostatic interactions among the permanently charged quaternary ammonium groups of TMC, the anionic groups of alginate/dextran sulfate, and charged residues of insulin. Similar behavior has been described for chitosan- and TMC-based insulin nanocarriers, where insulin is not only physically entrapped but also participates in polyelectrolyte complex formation [40,41]. Conversely, poloxamer^®^ exhibited an inverse effect on EE, suggesting that higher surfactant levels may reduce insulin association with the ADS-TMC complex. This effect may be related to steric shielding of interacting polymer chains or to increased solubilization of non-associated insulin in the external aqueous phase, thereby decreasing the fraction retained in the nanoparticles. Therefore, although poloxamer^®^ can contribute to colloidal stabilization, its concentration must be carefully controlled to avoid compromising insulin encapsulation. Overall, the screening design showed that the ADS-TMC pair is the main formulation lever controlling particle size, whereas TMC and poloxamer^®^ are the most influential variables for encapsulation efficiency, with opposite effects. These results justified selecting ADS and TMC concentrations for the subsequent optimization step and introducing the degree of quaternization of TMC as an additional factor, since TMC charge density is expected to influence both nanoparticle structure and mucoadhesive performance.

### 2.6. Response Surface Optimization and Desirability Analysis

The response-surface optimization was performed only for the CQAs selected in the Box–Behnken design, namely particle size, zeta potential, and in vitro mucoadhesion. Insulin release was not included as a modeled response and was instead evaluated separately as a complementary proof-of-concept functional parameter.

**Figure A2:**
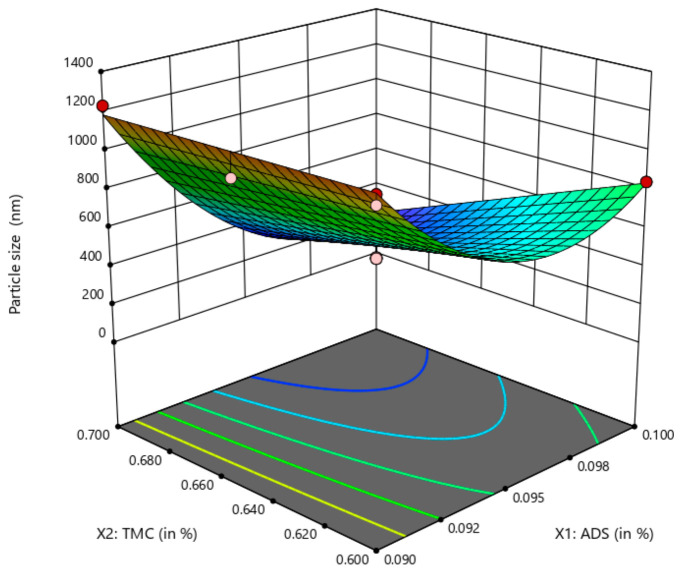
Response surface plot representing the effect of ADS (X1) and TMC (X2) concentrations on nanoparticle size (Y1).

**Figure A3:**
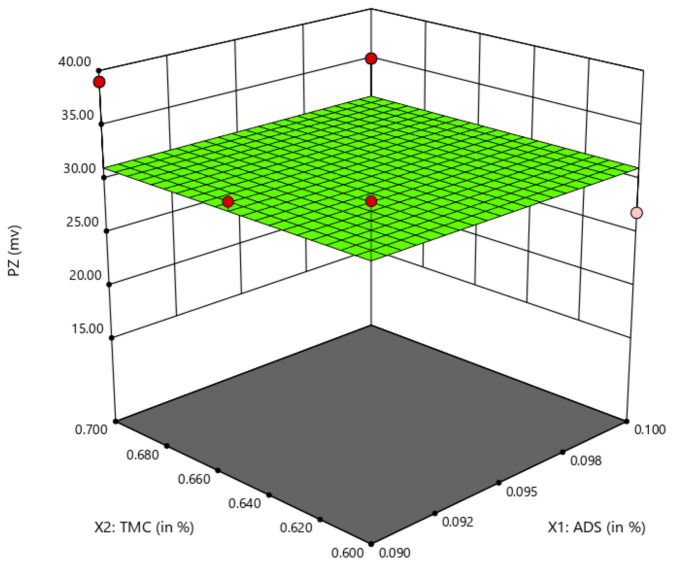
Response surface plot representing the effect of ADS (X1) and TMC (X2) concentrations on the nanoparticle’s potential zeta (Y2).

**Figure A4:**
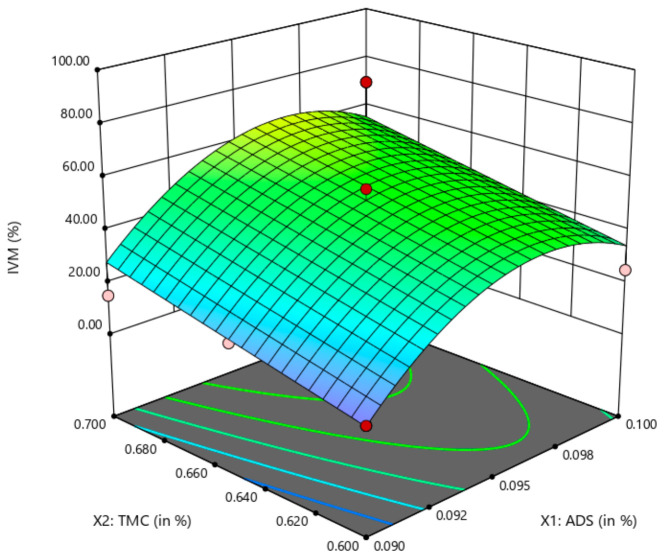
Response surface plot representing the effect of ADS (X1) and TMC (X2) concentrations on nanoparticle in vitro mucoadhesion (IVM) (Y3).

The relationships between the selected material attributes and the critical quality attributes (CQAs) were evaluated using response-surface models generated from the Box–Behnken design. ADS concentration, TMC concentration, and TMC degree of quaternization (DQ) were used as independent variables, while particle size (Y1), zeta potential (Y2), and in vitro mucoadhesion (Y3) were considered as responses. Linear or quadratic polynomial models were fitted to the experimental data, and model adequacy was assessed by ANOVA, lack-of-fit analysis, and regression statistics. The statistical parameters for each response model and response surface plot representing the effect of independent variables on each critical quality attribute are provided in Table A1 and Figure A2, Figure A3 and Figure A4 of the data in Appendix A. Coating pregel ADS cores with TMC under the conditions explored in the optimization design yielded nanoparticles with sizes ranging from 316 to 1340 nm, zeta potentials between +17 and +39 mV, and in vitro mucin binding ranging from 7 to 87%. Lower ADS concentrations (0.090% *w*/*v*) produced large, weakly mucoadhesive aggregates (1076–1340 nm, 7–16% mucin binding), whereas small increases in ADS to 0.095–0.100% *w/v* reduced particle size into the sub-micron range (316–845 nm) and markedly enhanced mucoadhesion (45–52%). This threshold behavior is consistent with polyelectrolyte-complex theory and with reports on alginate/chitosan systems showing that an adequate anionic polymer content is required to form compact nanostructures; too low an alginate content leads to loosely crosslinked aggregates rather than discrete nanoparticles [42,43]. However, identifying the optimal formulation factors for polycationic TMC/polyanionic ADS complexation to achieve higher mucoadhesion is challenging, and a multifactorial approach may be required. The best formulation (ADS 0.095% *w*/*v*, TMC 0.70% *w*/*v*, DQ 60%) combined the smallest size (316 nm) with a ζ-potential of approximately +38 mV and a maximum mucin-binding capacity (87%), thereby identifying a favorable composition for obtaining compact and highly mucoadhesive TMC coatings. Among the TMC-related factors, both TMC concentration and degree of quaternization (DQ) strongly affect CQAs particle size, zeta potential, and in vitro mucoadhesion. Increasing TMC from 0.60 to 0.70% *w/v* decreased average size (from 890 to 530 nm) and increased mucin binding (from 30 to 52%), provided ADS was not too low; at ADS ≥ 0.095% *w*/*v*, high TMC levels induced evident particle compaction and stronger mucin binding (e.g., 0.10% ADS, 0.70% TMC, DQ 40%, size of 345 nm, 70% mucoadhesion). A strong effect of DQ was observed. Increasing DQ from 20% to 60% increased mean ζ potential from +23 to +38 mV and nearly tripled average mucin binding (25 to 69%), while reducing mean size from 765 to 497 nm. The formulation with ADS 0.095%/TMC 0.70%/DQ 60% illustrates this three-factor combination, giving the most positively charged, smallest, and most mucoadhesive particles. This pattern is consistent with analyses of TMC chemistry literature data, in which higher quaternization increases positive charge density and mucoadhesion, thereby strengthening electrostatic interactions with negatively charged mucins [44,45]. The in vitro mucoadhesion results are consistent with the current understanding of mucoadhesive chitosan derivatives. Strongly cationic derivatives such as TMC exhibit enhanced mucoadhesion and permeation-enhancing ability at intestinal pH, because they remain protonated where chitosan would otherwise precipitate [46,47,48]. TMC-coated PLGA insulin nanoparticles (TMC PLGA NPs) displayed significantly increased retention in intestinal mucus and improved Caco 2 permeability compared with unmodified PLGA NPs, confirming that a TMC-rich, positively charged surface favors both mucoadhesion and epithelial interaction [49]. In the same way, self-assembled TMC insulin nanocarriers efficiently permeated mucus and opened tight junctions, leading to enhanced oral insulin absorption [41]. TMC-coated ADS nanoparticles, with a ζ potential range (+30 to +40 mV) and high mucin binding (up to 87%) in the best formulations, are within the “strongly mucoadhesive” class of TMC-based nanoparticles in these studies. Numerical optimization was then performed using the desirability function. The optimization goals were defined according to the intended quality profile of the nanoparticles: particle size was minimized, zeta potential was targeted toward sufficiently positive values compatible with aqueous colloidal stability, and in vitro mucoadhesion was maximized. The highest desirability was obtained for a formulation containing 0.096% (*w*/*v*) ADS and 0.700% (*w*/*v*) TMC with 60% DQ. Under these conditions, the model predicted a particle size of 316.24 nm, a zeta potential of +38.43 mV, and an in vitro mucoadhesion of 87.14%. The optimized formulation identified by desirability analysis should therefore be interpreted as the best compromise among the three modeled CQAs within the experimental factor space investigated in this study, rather than as a universal optimum for all possible formulation properties.

### 2.7. Optimization and Model Validation

The optimum nanoparticle formulation was selected by numerical desirability analysis using the constraints established for the Box–Behnken design. The optimized formulation consisted of 0.096% (*w*/*v*) ADS and 0.700% (*w*/*v*) TMC with a TMC degree of quaternization of 60%. Under these conditions, the model predicted a particle size of 316.24 nm, a zeta potential of +38.43 mV, and an in vitro mucoadhesion of 87.14%. Experimental confirmation yielded values of 330.79 nm, +37.09 mV, and 84.61%, respectively, corresponding to prediction errors of 4.6%, 3.5%, and 2.9% (Table 2). Since all prediction errors were below 5%, the optimization model was considered validated for the selected formulation. The good correspondence between predicted and experimental responses supports the robustness of the optimization strategy and demonstrates the usefulness of the QbD approach for guiding formulation development. In practical terms, the optimized TMC-coated ADS nanoparticles retained the relevant attributes considered for oral insulin delivery, namely a submicron size range, a positive zeta potential compatible with aqueous stability, and high in vitro interaction with mucin. Taken together, the confirmation results validate the use of the fitted response-surface models and the desirability function as reliable tools for selecting formulation conditions for mucoadhesive ADS/TMC nanoparticles.

## 3. Materials and Methods

### 3.1. Materials

Alginic acid sodium salt (200 kDa with a mannuronic/guluronic ratio of 1.56), 87 KDa chitosan (CHI) with a deacetylation degree of 85%, and trifluoroacetic acid 99% (*v*/*v*) were purchased from Sigma-Aldrich (Madrid, Spain), dextran sulfate sodium salt (5 kDa) and polyvinylpyrrolidone (PVP) K 30 were purchased from Fluka (Buchs, Switzerland), poloxamer^®^ 188 (Lutrol^®^ F68) was kindly supplied by BASF (Hürth, Germany), calcium chloride and sodium chloride were purchased from Riedel-de-Haën (Lower Saxony, Germany), lactic acid 90% was purchased from VWR BDH Prolabo (Rosny-sous-Bois, France), polyethylene glycol 4000 (PEG 4000) was acquired from Fisher Scientific^®^ (Loughborough, UK), acetonitrile LiChrosolv^®^, hydrochloric acid 37%, potassium dihydrogen phosphate and sodium hydroxide were purchased from Merck KGaA (Darmstadt, Germany), and Actrapid^®^ 100 IU/mL (Novo Nordisk A/S, Bagsværd, Denmark) was supplied by a local pharmacy. Chitosan was dissolved in an aqueous solution containing 0.5% lactic acid (*v*/*v*) and vacuum-filtered through a Millipore#2 paper filter. Cellulose membrane with a tubing nominal dry thickness of 10 kDa molecular weight cutoff (MWCO) (SnakeSkin Pleated Dialysis Tubing) and dialysis diffusion bags (Spectra/Por^®^, with an MWCO of 100 kDa were purchased from Thermo Fisher Inc. (Waltham, MA, USA) and Biotech CE, Spectrum Laboratories Inc. (Piscataway, CA, USA), respectively. Materials used in trimethyl chitosan (TMC) synthesis, such as sodium hydroxide, sodium iodide, sodium chloride, sodium acetate, acetic acid, hydrochloric acid, N-methyl-2-pyrrolidinone (NMP), potassium dihydrogen phosphate, ethanol, and diethyl ether, were purchased from Sigma-Aldrich and were of analytical grade unless otherwise stated.

### 3.2. Methods

#### 3.2.1. Synthesis of TMC

TMC samples with three degrees of quaternization (DQ = 20 mol%, DQ = 40 mol%, and DQ = 60 mol%) were synthesized and are referred to as TMC20, TMC40, and TMC60, respectively. TMC20 was obtained using a single-step quaternization procedure, whereas TMC40 and TMC60 were produced by applying an additional methylation step based on the multi-step strategy reported for achieving higher substitution of CHI.

##### Preparation of TMC20

In an adaptation of a published technique [30], CHI (10 g) was dissolved in NMP (1/4) (*w*/*v*) at 45 °C under magnetic stirring for approximately 30 min. Sodium iodide (NaI; 24 g) and aqueous NaOH (15% *w*/*v*; 60 mL) were then added, and the mixture was stirred for an additional 20 min. The reaction system was maintained under reflux, and methyl iodide (55 mL) was added; the reaction was allowed to proceed for 1 h at 45 °C. The resulting TMC iodide reaction solution was isolated by precipitation in ethanol (1/3) (*v*/*v*). The precipitate was collected by centrifugation (3000 rpm, 5 min), washed with ethanol (four washes) and diethyl ether (three washes), filtered, and dried under vacuum at 40 °C for 48 h.

To obtain the chloride form, the dried polymer was dissolved in 10% (*w*/*v*) aqueous NaCl to exchange iodide for chloride. The resulting TMC chloride was reprecipitated in ethanol and recovered by centrifugation using the same isolation approach described above. The material was stored protected from light until further use.

##### Preparation of TMC40 and TMC60

A two-step quaternization strategy was adapted from a published technique [30], in which the second methylation step follows another reported technique [50]. An iodide TMC intermediate was first synthesized from chitosan (10 g) dissolved in NMP (1/4)/*w*/*v* at 45 °C, followed by the addition of NaI (24 g) and aqueous NaOH (15% *w*/*v*; 60 mL). Methyl iodide (55 mL) was then added under reflux, and the reaction proceeded for 1 h at 45 °C. The iodide TMC intermediate was recovered by ethanol precipitation, washed, and dried.

For the second step, the dried iodide-TMC intermediate was redissolved in NMP under constant stirring and subjected to a second methylation by sequential addition of NaI, aqueous NaOH, and methyl iodide under rapid stirring, followed by further additions of methyl iodide and NaOH, and continued stirring for 1 h. The product was precipitated in ethanol, washed, filtered, and dried. Finally, iodide counterions were exchanged to chloride by dissolving the product in 10% (*w*/*v*) NaCl, reprecipitating in ethanol, and isolating the chloride form by centrifugation. TMC40 and TMC60 were obtained upon adjusting the methyl iodide charge, and the achieved DQ values were confirmed by ^1^H NMR. The material was stored protected from light until use.

#### 3.2.2. Preparation of Nanoparticles

Nanoparticles were prepared through a two-step process consisting of ADS pre-gel formation followed by TMC complexation. Briefly, an aqueous phase at pH 4.6 containing sodium alginate, dextran sulfate, poloxamer^®^ 188, and insulin was maintained under stirring while calcium chloride solution was added dropwise using a peristaltic pump. This step generated pre-gelled ADS cores through ionic crosslinking. The coating phase, containing TMC and PEG 4000 at pH 7.0, was then slowly introduced into the core dispersion to promote electrostatic complexation between the polyanionic ADS matrix and the cationic TMC chains. The resulting nanoparticles were concentrated by dialysis against PVP K30 solution at 4 °C [13]. After concentration, the suspension pH was adjusted to 7.0, and KNO_3_ was added as an ionic stabilizer [6].

#### 3.2.3. Physicochemical Characterization

##### Intrinsic Viscosity and Viscosity Average Molecular Weight (Mv)

The intrinsic viscosities [η] of TMCs were determined in an acetic acid/sodium acetate buffer following the capillary viscometry procedure previously described [51]. Measurements were carried out using a Ubbelohde-type capillary viscometer Model 100/E534 (Cannon Instrument Company, State College, PA, USA, Instrumentación Analitica S.A., Barcelona, Spain) maintained at 25 °C. Intrinsic viscosity data were used to estimate the viscosity average molecular weight (Mv) via the Mark–Houwink–Sakurada relationship, applying constants reported for TMC with an acetylation degree of 15 mol% (K = 1.38 × 10^−5^ and a = 0.85) [51]. Using these parameters, the calculated Mv values were 29 × 10^3^ g·mol^−1^ for TMC20, 23 × 10^3^ g·mol^−1^ for TMC40, and 16 × 10^3^ g·mol^−1^ for TMC60.

##### Granulometric Size Distribution

Particle size was measured by dynamic light scattering using a NanoZetasizer at 25 °C and a backscattering angle of 173°. Samples were diluted according to the validated operating range of the instrument, and six measurements were collected for each formulation. The acquisition settings were automatically adjusted by the software to satisfy measurement-quality criteria. Intensity-weighted distributions were used to report particle diameter, whereas number-weighted distributions were calculated by the software to estimate the relative contribution of different particle populations. Short-term aqueous stability was evaluated by repeating size measurements after storage at 2–8 °C [6].

##### Zeta Potential Analysis

Zeta potential, reflecting the electrical charge at the nanoparticle surface, was measured using the same instrument. For each sample, three automated determinations were performed.

##### Encapsulation Efficiency (EE)

Encapsulation efficiency was determined indirectly by quantifying the insulin remaining in the supernatant after separation of nanoparticles from the non-associated peptide. Samples were centrifuged at 20,000× *g* for 60 min at 4 °C, and the supernatants were collected and mixed with the mobile phase before HPLC analysis. Insulin was quantified in triplicate, and EE was calculated from the difference between the total insulin added to the formulation and the free insulin detected in the supernatant. Nanoparticle dispersions were subsequently frozen at −80 °C, lyophilized, and stored in a desiccator until constant mass was reached.

##### Insulin Release Studies

Release experiments were conducted in enzyme-free media in order to assess the pH-responsive behavior of the nanoparticles without the interference effects of enzymatic degradation. Nanoparticles were first incubated in simulated gastric medium at pH 1.2 for 120 min and then transferred to simulated intestinal medium at pH 6.8, thereby allowing a clearer evaluation of the pH transition from gastric to intestinal conditions. At each sampling point, the collected aliquots were centrifuged (20,000× *g* for 15 min), and the insulin concentration in the supernatant was determined by HPLC. Cumulative insulin release was expressed as the percentage of insulin released from the nanoparticles over the course of the experiment. All release assays were performed in triplicate.

##### Insulin Determination

Insulin quantification was carried out on an LC-2010 HT HPLC system (Shimadzu Co., Kyoto, Japan) equipped with a quaternary pump and an HP 1050 programmable multi-wavelength UV detector set at 214 nm. Separation was achieved on a reversed-phase X-Terra^®^ RP-18 column (5 µm, 4.6 × 250 mm; Waters Co., Milford, MA, USA) preceded by a Purospher STAR^®^ RP-18 guard column (5 µm; Merck KGaA, Darmstadt, Germany). The mobile phase consisted of acetonitrile (A) and an aqueous solution of 0.1% (*v*/*v*) trifluoroacetic acid (B), delivered at 1.0 mL/min under a gradient program starting at 30:70 (A:B), changing to 40:60 (A:B) over 5 min (with a 5 min elution at this composition), and then returning to 30:70 (A:B) within 1 min (followed by a 1 min elution). Chromatographic peak areas were integrated automatically, and the method was validated over the concentration range 2.1–108 µg/mL, with a correlation coefficient of R^2^ = 0.9996.

##### Conformational Stability of Insulin

The structural integrity of insulin after nanoparticle release was examined by circular dichroism (CD) spectroscopy. Spectra were recorded at 25 °C using a Jasco J-815 spectropolarimeter (Jasco, Lisses, France) equipped with temperature control and a 0.1 cm path-length cuvette. Measurements were performed from 200 to 260 nm using a 0.2 nm resolution, a scan rate of 50 nm/min, and a response time of 4 s. Each spectrum was obtained by averaging five scans after subtraction of the corresponding buffer blank. Released insulin samples prepared in PBS were compared with unprocessed insulin at the same concentration.

##### Fourier-Transform Infrared Analysis

All samples for FTIR analysis were prepared as described for the encapsulation efficiency experiments. Infrared spectra of the raw materials and of the final nanoparticle formulations were recorded using a 400N FT-NIR Imaging System (Perkin-Elmer, Waltham, MA, USA). For each sample, 64 scans were accumulated at a spectral resolution of 4 cm^−1^ over the 750–3750 cm^−1^ wavenumber range.

##### In Vitro Mucoadhesion Assay

The ability of nanoparticles to bind mucin was assessed using an indirect in vitro assay. Equal volumes of mucin solution and nanoparticle suspension were mixed and incubated at 37 °C for 2 h under agitation. After incubation, mucin-associated nanoparticles were separated by centrifugation (20,000× *g*/40 min), and the amount of unbound mucin remaining in the supernatant was quantified using Bradford reagent (Sigma-Aldrich, Madrid, Spain). Absorbance was measured at 620 nm, and mucin concentration was calculated from a calibration curve prepared under the same conditions.

#### 3.2.4. Experimental Design

##### Exploratory Experiments

The stirring speed and the polycation addition flow rate during the coating of ADS pregel-state cores are processing parameters that may affect particle size. To further limit the number of parameters to study in the DoE, the processing parameters were varied in exploratory experiments to determine their optimal values.

Stirring speed was varied from 800 to 1400 rpm using a magnetic oblong bar (12 mm width × 5 mm height) in a 600 mL recipient. A flow rate ranging from 1 mL/min to 2.0 mL/min, monitored through a peristaltic pump, was used to add polycation solution to the ADS cores- containing medium. The resulting particles were characterized for size and PDI.

##### Assessment of Factor Criticality Using a Screening Design

Using a full factorial design, a first run of experiments was conducted to estimate and rank four independent factors: ADS concentration (x1), TMC concentration (x2), insulin concentration (x3), and poloxamer^®^ concentration (x4). The objective is to improve understanding of the criticality of factors often considered material attributes. The ranges of anionic biopolymers ADS, insulin, and poloxamer^®^ concentrations were established based on previous studies of similar insulin-containing nanoparticles [6,13]. For the polycation, TMC solutions at 0.3% and 0.6% (*w*/*v*) were used, based on literature data and experience on chitosan complexation with polyanions, alginate, and dextran sulfate. Process parameters such as stirring speed and polycation addition flow rate were set at 1300 rpm and 1.6 mL/min, respectively. The influence of these factors (×1–×4) was examined using a two-level test, with low (−1) and high (+1) levels as shown in Table 3. The dependent responses were particle size (y1) and encapsulation efficiency (y2). Statistical evaluation of the influence of the four input factors on the two critical quality attributes was performed by fitting a linear regression model to the qualitative factor levels.

##### Response Surface Optimization of the Formulation by Box–Behnken Design

**Figure A5:**
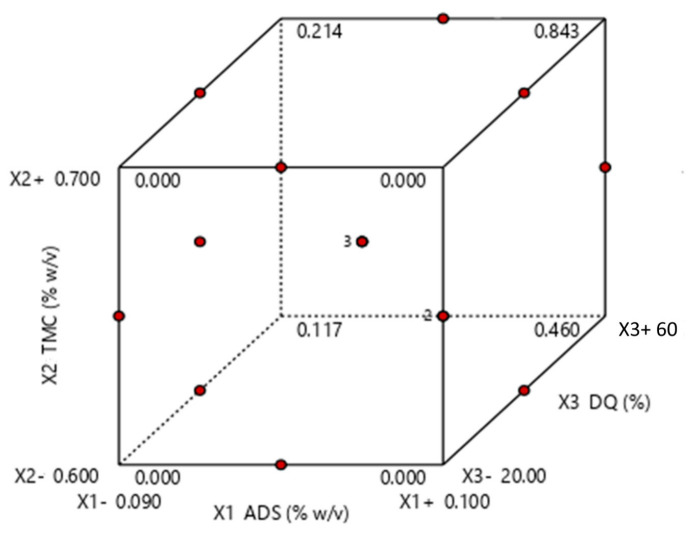
Three-dimensional representation of the Box–Behnken design used for formulation optimization.

Based on the screening results, ADS concentration and TMC concentration were selected for response-surface optimization. The degree of quaternization of TMC was introduced as an additional material attribute because it directly affects the density of permanent positive charges on the polymer chain. A three-factor, three-level Box–Behnken design was then used to evaluate the influence of ADS concentration, TMC concentration, and DQ on particle size, zeta potential, and in vitro mucoadhesion. represented in Table 4, was implemented (the cube representation of the design is displayed in Figure A5 in Appendix A). The Box–Behnken design is a class of response-surface designs in which second-order polynomial models are fitted to experimental data, making it particularly suitable for optimization of response variables and identifying optimal material attributes and process parameters [23,52].

The experimental responses were fitted to polynomial models, and the adequacy of each model was evaluated using ANOVA and the regression statistics generated by Design-Expert^®^.

Numerical optimization was performed using the desirability function, with the goals of minimizing particle size, obtaining a sufficiently positive zeta potential compatible with aqueous colloidal stability, and maximizing in vitro mucoadhesion. The predicted optimum was prepared independently, and the observed responses were compared with the predicted values. Model confirmation was accepted when the relative prediction error for each response was below 5%.

#### 3.2.5. Model Validation

For each response, the experimental data generated by the Box–Behnken design were fitted to linear or quadratic polynomial models in Design-Expert^®^ software 13 (Stat-Ease, Minneapolis, MN, USA). Model adequacy was assessed by analysis of variance (ANOVA), taking into account model, lack-of-fit test, and the regression statistics provided by the software, including the coefficient of determination (R^2^), adjusted R^2^, and predicted R^2^. Detailed statistical parameters for the fitted models are provided in Table A1. Numerical optimization was performed in Design-Expert^®^ using the desirability function. The optimization goals were defined according to the intended quality profile of the nanoparticles, namely minimization of particle size, attainment of a sufficiently positive zeta potential compatible with aqueous colloidal stability, and maximization of in vitro mucoadhesion. The optimized formulation was selected as the factor combination giving the highest overall desirability while satisfying these predefined criteria.

The optimum nanoparticle formulation was selected by numerical desirability analysis using the constraints established for the Box–Behnken design. The optimized formulation consisted of 0.096% (*w*/*v*) ADS and 0.700% (*w*/*v*) TMC with a TMC degree of quaternization of 60%. Under these conditions, the model predicted a particle size of 316.24 nm, a zeta potential of +38.43 mV, and an in vitro mucoadhesion of 87.14%. Experimental confirmation yielded values of 330.79 nm, +37.09 mV, and 84.61%, respectively, corresponding to prediction errors of 4.6%, 3.5%, and 2.9%. Since all prediction errors were below 5%, the optimization model was considered validated for the selected formulation.

#### 3.2.6. Statistical Analysis

Data are presented as mean ± standard deviation (SD) from at least three independent experiments. Statistical comparisons were performed by one-way analysis of variance (ANOVA) followed by Bonferroni’s post hoc test using SPSS 20.0 (IBM, Chicago, IL, USA). Differences were considered statistically significant at *p* < 0.05, with higher levels of significance indicated as *p* < 0.01 and *p* < 0.001.

## 4. Conclusions

TMC-coated ADS nanoparticles were successfully developed as mucoadhesive carriers for oral insulin delivery using a QbD-guided workflow. Screening experiments showed that the ADS-TMC balance was the main determinant of particle size, while TMC and poloxamer^®^ concentrations were the most relevant factors affecting encapsulation efficiency. Subsequent Box–Behnken optimization demonstrated that ADS concentration, TMC concentration, and TMC DQ controlled particle size, zeta potential, and in vitro mucoadhesion. Numerical optimization by Design-Expert^®^ desirability analysis selected an optimized formulation containing 0.096% (*w*/*v*) ADS and 0.700% (*w*/*v*) TMC with 60% DQ. The optimized formulation was experimentally confirmed, yielding a particle size of 330.79 nm, a zeta potential of +37.09 mV, and in vitro mucoadhesion of 84.61%. These values were close to the model predictions, with prediction errors below 5% for all responses, confirming the predictive ability of the fitted response-surface models at the selected formulation conditions. Overall, the results demonstrate that a QbD strategy combined with desirability-based optimization is a useful tool for rationally developing TMC-coated ADS nanoparticles with suitable physicochemical and mucoadhesive properties for oral insulin delivery. A limitation of the present study is the absence of direct microscopic visualization of the nanoparticles. Although DLS, laser diffraction, zeta potential, FTIR, and mucoadhesion results support the formation of nanoscale ADS/TMC polyelectrolyte complexes, future work should include microscopy-based characterization to confirm particle morphology and structural organization. Future studies should extend this framework to additional functional outcomes, including insulin release under biorelevant conditions and in vivo pharmacodynamic evaluation.

## Figures and Tables

**Table 1:** Experimental results of the two-level screening design evaluating the effects of ADS concentration (x1), TMC concentration (x2), insulin concentration (x3), and poloxamer^®^ concentration (x4) on particle size and encapsulation efficiency.

x1% (*w*/*v*)	x2% (*w*/*v*)	x3% (*w*/*v*)	x4% (*w*/*v*)	Particle Size (nm)	Encapsulation Efficiency (%)
0.05	0.30	0.008	0.06	277	68
0.10	0.60	0.004	0.06	378	54
0.10	0.30	0.008	0.03	218	73
0.10	0.60	0.008	0.03	418	47
0.05	0.30	0.004	0.06	437	46
0.05	0.30	0.004	0.03	437	72
0.05	0.60	0.008	0.06	473	48
0.05	0.60	0.004	0.03	457	45
0.10	0.30	0.008	0.06	378	73
0.05	0.60	0.008	0.03	468	49
0.10	0.30	0.004	0.03	404	69
0.05	0.60	0.004	0.06	448	47
0.05	0.30	0.008	0.03	224	81
0.10	0.30	0.004	0.06	427	67
0.10	0.60	0.004	0.03	357	44
0.10	0.60	0.008	0.06	238	84

**Table 2:** Comparison between predicted and experimental responses and predicted error for optimized nanoparticle formulation.

Dependent Variable	Predicted Value	Experimental Value	Predicted Error (%)
Particle size (nm)	316.24	330.79	4.6
Zeta potential (mV)	38.43	37.09	3.5
In vitro mucoadhesion (%)	87.14	84.61	2.9

**Table 3:** Independent and dependent variables in the 2^4^ full factorial screening design for nanoparticle formulation. Each of the 16 experimental conditions was carried out in duplicate.

Independent Variables		Level		Unit
		−1	+1	
x1	ADS	0.05	0.10	% (*w*/*v*)
x2	TMC	0.30	0.60	% (*w*/*v*)
x3	Insulin	0.004	0.008	% (*w*/*v*)
x4	Poloxamer^®^	0.03	0.06	% (*w*/*v*)
Dependent variables				Unit
y1	Particle size			nm
y2	Encapsulation efficiency			% (*w*/*w*)
Run	x1% (*w*/*v*)	x2% (*w*/*v*)	x3% (*w*/*v*)	x4% (*w*/*v*)
1	0.05	0.30	0.008	0.06
2	0.10	0.60	0.004	0.06
3	0.10	0.30	0.008	0.03
4	0.10	0.60	0.008	0.03
5	0.05	0.30	0.004	0.06
6	0.05	0.30	0.004	0.03
7	0.05	0.60	0.008	0.06
8	0.05	0.60	0.004	0.03
9	0.10	0.30	0.008	0.06
10	0.05	0.60	0.008	0.03
11	0.10	0.30	0.004	0.03
12	0.05	0.60	0.004	0.06
13	0.05	0.30	0.008	0.03
14	0.10	0.30	0.004	0.06
15	0.10	0.60	0.004	0.03
16	0.10	0.60	0.008	0.06

**Table 4:** Dependent and independent variables in a 3^3^ Box–Behnken design of nanoparticle formulations. Each of the 15 runs was performed in triplicate.

		Level	
Independent variables	−1	0	1
ADS % (*w*/*v*)	0.09	0.095	0.100
TMC % (*w*/*v*)	0.60	0.65	0.70
DQ %	20	40	60
Dependent variables	Constraint		
Particle size (nm)	Minimize		
Zeta potential (mV)	≥+30 mV		
In vitro mucoadhesion (%)	Maximize		
Run	X1% (*w*/*v*)	X2% (*w*/*v*)	X3% (*w*/*v*)
1	0.090	0.600	40
2	0.100	0.600	40
3	0.090	0.700	40
4	0.100	0.700	40
5	0.090	0.650	20
6	0.100	0.650	20
7	0.090	0.650	40
8	0.100	0.650	60
9	0.095	0.600	20
10	0.095	0.700	20
11	0.095	0.600	60
12	0.095	0.700	60
13	0.095	0.650	40
14	0.095	0.650	40
15	0.095	0.650	40

## Data Availability

The original contributions presented in this study are included in the article.

## Abbreviations

ADS	alginate/dextran sulfate
ANOVA	analysis of variance
CD	circular dichroism
CQA	critical quality attribute
DLS	dynamic light scattering
DQ	degree of quaternization
EE	encapsulation efficiency
FTIR	Fourier-transform infrared spectroscopy
HPLC	high-performance liquid chromatography
IVM	in vitro mucoadhesion
NMR	nuclear magnetic resonance
PBS	phosphate-buffered saline
PDI	polydispersity index
PEG	polyethylene glycol
QbD	Quality by Design
TMC	trimethyl chitosan

## Appendix A

**Table A1:** Estimate of the coefficients of the regression equation for formulation factors as independent variables, standard error, and fit statistics.

Coefficient		Y_1_			Y_2_			Y_3_	
	C	SE	Range *	C	SE	Range *	C	SE	Range *
$b_{0}$	513.14	38.11	428.23 to 598.05	31.16	1.07	28.85 to 33.48	51.71	3.82	43.20 to 60.23
$b_{1}$	−332.63	35.65	−412.05 to −253.20	-	-	-	13.81	3.61	5.78 to 21.85
$b_{2}$	−160.00	35.65	−239.42 to 80.58	-	-	-	10.63	3.57	2.66 to 18.59
$b_{3}$	-	-	-	7.45	1.57	4.06 to 10.84	17.48	3.89	8.81 to 26.15
${b_{1}b}_{2}$	140.50–140.50	50.41	−252.82 to −28.18	-	-	-		-	-
${b_{1}b}_{3}$	-	-	-	-	-	-	-	-	-
${b_{2}b}_{3}$	-	-	-	-	-	-	-	-	-
${b_{1}}^{2}$	357.73	52.18	241.47 to 473.99	-	-	-	−20.53	5.25	−32.23 to −8.82
${b_{2}}^{2}$	-	-	-	-	-	-	-	-	-
${b_{3}}^{2}$	-	-	-	-	-	-	-	-	-
Fit statistics									
R^2^		0.9418598163235			0.63394515747699			0.87169805609252	
Adjusted R^2^		0.9186037428529			0.60578709266753			0.82037727852953	
Predicted R^2^		0.84764188538136			0.53248331059176			0.67241475401584	

C: coefficient estimate; SE: standard error. * The range indicates the lower and upper values of coefficients at 95% confidence interval.
